# Charlson comorbidity index and 1-year poor outcomes in elderly patients undergoing successful percutaneous coronary intervention: A retrospective study

**DOI:** 10.1097/MD.0000000000033792

**Published:** 2023-05-12

**Authors:** Ahmet Balun, Alkame Akgümüş

**Affiliations:** a Bandirma Onyedi Eylül University, Department of Cardiology, Balikesir, Turkey.

**Keywords:** acute coronary syndrome, Charlson comorbidity index, comorbidity, elderly

## Abstract

Elderly patients with acute syndrome are frailer due to the burden of comorbidity. Comorbidities that increase with age result in an increased risk of mortality in patients with acute coronary syndrome (ACS). Many scales have been developed to assess the burden of comorbidity, including the Charlson Comorbidity Index (CCI). The aim of our study is to show the effect of the CCI on 1-year mortality and poor clinical outcomes in elderly patients who underwent percutaneous coronary intervention due to ACS. This single-center retrospective study included 704 patients aged 75 years and older. The study population consisted of patients who were admitted to the hospital with ACS between April 2017 and September 2021 and underwent successful percutaneous intervention. The patients were divided into 3 groups according to their CCI scores as CCI 0 (n:156), 1 (n:266), and ≥2 (n:282). Stroke development was significantly higher in patients with CCI scores ≥ 2 compared to the other 2 groups (*P* = .005). Mortality rates were found to be 28.4%, 7.5%, and 2.6% in patients with CCI ≥ 2, CCI 1, and CCI 0, respectively. The mortality rate of the CCI ≥ 2 group was significantly higher than those of the other 2 groups (*P* < .001). The multivariate Cox proportional hazard regression model showed that CCI was an independent predictor for 1-year all-cause mortality (hazard ratio: 1.632; 95% confidence interval: 1.403–1.898; *P* < .001). CCI may contribute to treatment and follow-up management, as it indicates a poor prognosis in elderly patients who have undergone percutaneous coronary intervention.

## 1. Introduction

The incidence of ischemic heart disease is increasing along with the aging of the world population, and it is the leading cause of death globally. Aging causes patients to be physically weaker and frailer. Therefore, old age is an independent predictor of poor prognosis in patients with acute coronary syndrome (ACS).^[1]^ Comorbidities that increase with age result in an increased risk of mortality in patients with ACS.^[2]^ Clinical decision-making and treatment are difficult in elderly patients with ACS because of the increased risk of mortality and additional cardiovascular events due to the burden of comorbidity.

Comorbid conditions that pose a risk for the development of cardiovascular disease should be considered as a whole. Elderly patients usually have more than 1 comorbid condition. Thus, many scales have been developed to assess the burden of comorbidity, including the Charlson Comorbidity Index (CCI). Previous myocardial infarction, congestive heart failure, peripheral arterial disease, cerebrovascular accident or transient ischemic attack, dementia, chronic obstructive pulmonary disease, connective tissue disorder, peptic ulcer disease, liver disease, diabetes, hemiplegia, chronic kidney disease, malignant neoplasm, leukemia and lymphoma, metastatic solid tumor, and AIDS are comorbid conditions included in the CCI. Each condition is scored between 1 and 6 based on its prognostic value. The CCI has previously been used to predict prognosis in diseases such as ACS, heart failure, cerebrovascular disease, and cancer.^[3–6]^ The aim of our study is to show the effect of the CCI on 1-year mortality and poor clinical outcomes in elderly patients who underwent percutaneous coronary intervention after ACS.

## 2. Methods

This single-center retrospective study included 704 patients aged 75 years and older. The study population consisted of patients who applied to the Bandirma Training and Research Hospital with ACS between April 2017 and September 2021 and underwent successful percutaneous intervention. Clinical and laboratory results, demographic data, and current comorbidity status were obtained from the hospital archive and electronic registry system. This study was performed in line with the principles of the Declaration of Helsinki. Approval was granted by the Ethics Committee of Bandirma Onyedi Eylül School of Medicine, approval number 2022/7-1 and dated 19.08.2022. Since the study was conducted in a center with 24-hour coronary angiography facilities, early percutaneous coronary intervention was performed in all of the patients. Patients who had regular cardiology outpatient visits and received dual antiplatelet therapy until the end of the follow-up period were included in the study. Patients using oral anticoagulants were excluded from the study, as they may affect clinical outcomes. Other exclusion criteria were patients who did not undergo percutaneous coronary intervention, patients who were recommended coronary bypass surgery after coronary angiography, and patients with in-hospital mortality.

CCI is a widely used death assessment model. The necessary information for CCI was obtained from the hospital registry system. Age was not evaluated in the CCI score as the study population consisted of elderly patients. All-cause mortality, which was the study endpoint, was obtained from the national electronic health record system. In addition, hospital admissions due to major and minor bleeding, cerebrovascular accident, and recurrent myocardial infarction were obtained from hospital records. Major and minor hemorrhages were determined according to the International Society on Thrombosis and Hemostasis criteria.^[7]^ Since the patient population consisted of patients over 75 years of age, it was expected that most of them would have at least 1 comorbidity. The patients were divided into 3 groups according to their CCI scores as CCI 0, 1, and ≥2.

In summarizing the data obtained from the study, descriptive statistics were tabulated as mean ± standard deviation or median, quartile 1, and quartile 3 depending on the distribution for continuous (numerical) variables. Categorical variables were summarized as numbers and percentages. The normality of numeric variables was checked with the Kolmogorov–Smirnov, test. Comparisons of the CCI groups were made using a 1-way analysis of variance when the numerical variables showed a normal distribution and a Kruskal–Wallis H test when they did not. Multiple comparisons were evaluated with the Tukey test when parametric tests were used and with the Dwass–Steel–Critchlow–Fligner test when non-parametric tests were used. Pearson chi-squared test was used for comparisons when there were 5 or more expected cells in the differences between the categorical variables according to the groups, and the Fisher–Freeman–Halton test was used for the tables when there were fewer than 5 expected cells. The association between CCI and 1-year all-cause mortality was analyzed using multivariate Cox proportional hazards regression models, and the results were expressed as hazard ratio and 95% confidence interval. The selection of variables for the multivariate analysis was based on previous reports and clinical importance. Kaplan–Meier survival analysis and a log-rank test were performed to compare 1-year all-cause mortality between the CCI groups. Statistical analyses were performed with the SPSS Version 22 for Windows (SPSS Inc, Chicago, IL), and the level of significance was considered to be .05 (*P* value).

## 3. Results

There were 704 patients in the study with a mean age of 82.4 ± 4.5 years. In terms of gender distribution, the study group consisted of 376 male patients (53.4%) and 328 female patients (46.6%). There were 156 (22.2%) patients in the CCI 0 group, 266 (37.8%) in the CCI 1 group, and 282 (40.0%) in the CCI ≥ 2 group. Demographics, clinical characteristics are summarized in Table 1. In terms of ACS diagnoses, non-ST elevation myocardial infarction was the most common diagnosis (380 patients, 54.0%).

**Table 1:** Demographic, clinical characteristics according to Charlson comorbidity index.

	Charlson Comorbidity Index (CCI)			
	CCI: 0 (n = 156)	CCI: 1 (n = 266)	CCI ≥ 2 (n = 282)	*P*
Age (yr)	79.8 ± 3.6	82.5 ± 4.6	83.7 ± 4.3	<.001
Sex, n (%)				
Male	86 (55.1)	148 (55.6)	142 (50.4)	.64
Female	70 (44.9)	118 (44.4)	140 (49.6)	
Body mass index (kg/m^2^)	27.3 ± 3.7	26.8 ± 2.5	26.9 ± 2.6	.57
Current smoker, n (%)	34 (21.8)	58 (21.8)	44 (15.6)	.35
Hypertension, n (%)	58 (37.2)	178 (66.9)	226 (80.1)	<.001
Hyperlipidemia, n (%)	18 (11.5)	40 (15.0)	68 (24.1)	.03
Previous CABG	4 (2.6)	18 (6.8)	42 (14.9)	.005
Previous PCI	18 (11.5)	44 (16.5)	62 (22.0)	.13
ACS presentation				
USAP	6 (3.8)	14 (5.3)	12 (4.3)	.66
NSTEMI	74 (47.4)	146 (54.9)	160 (56.7)	
STEMI	76 (48.7)	106 (39.8)	110 (39.0)	
GRACE score	146.2 ± 15.7	148.6 ± 14.6	147.1 ± 14.8	.51
Systolic blood pressure, mm Hg	120 [106–137]	128 [110–140]	125 [110–140]	.03
Dastolic blood pressure, mm Hg	67 [62–75]	70 [63–81]	70 [60.5–78]	.48
Heart rate, bpm	72 [68–84]	80 [70–89]	80 [71–91]	.01
LVEF, %	55 [50–60]	55 [50–60]	51 [45–60]	.03

CABG = coronary artery bypass graft, LvEF = Left ventricular ejection fraction, NSTEMI = non-ST-segment elevation myocardial infarction, PCI = percutaneous coronary intervention, STEMI = ST-segment elevation myocardial infarction, USAP = unstable angina pectoris.

Significant differences were found between the groups in terms of age and comorbidities. The patients in the CCI ≥ 2 group were older than the patients in the CCI 1 and CCI 0 groups (*P* < .001). There was a significant age difference between the CCI ≥ 2 and CCI 1 groups (*P* = .04). The groups were similar in terms of gender and Body Mass Index (*P* = .64 and *P* = .57). There were significant differences between the groups in terms of hypertension (*P* < .001), hyperlipidemia (*P* = .03), and previous coronary bypass surgery (*P* = .005). Patients with CCI ≥ 2 had significantly higher hypertension than patients in the CCI 1 and CCI 0 groups. The rate of hyperlipidemia was significantly higher in patients in the CCI ≥ 2 and CCI 1 groups than in patients in the CCI 0 group (*P* = .03). The rates of previous coronary bypass surgery were significantly higher in patients in the CCI ≥ 2 group than in patients in the other 2 groups (*P* = .005). There was no significant difference between the groups in terms of ACS presentations (*P* = .66). There was a significant difference between the groups in terms of median systolic blood pressure value, median heart rate and ejection fraction (*P* = .03, *P* = .01 and *P* = .03).

Laboratory parameters are summarized in Table 2. Significant differences were also found between the CCI ≥ 2 and CCI 0 groups with regard to hemoglobin values (*P* = .03). The groups were similar in terms of other variables (*P* > .05).

**Table 2:** Laboratory characteristics according to Charlson comorbidity index.

	Charlson Comorbidity Index (CCI)			
	CCI: 0 s(n = 156)	CCI: 1 (n = 266)	CCI ≥ 2 (n = 282)	*P*
Hemoglobin, g/dL	12.9 ± 1.6	12.8 ± 1.6	12.4 ± 1.8	.03
Lymphocyte count, per mm^9^	7.9 [6.9–11.9]	8.3 [6.8–10.5]	8.4 [6.8–11.3]	.21
Platelet count, per mm^3^	220 [182–272]	233 [180–275]	230 [190–279]	.66
Creatinine, mg/dL	1.0 [0.88–1.14]	0.96 [0.8–1.18]	1.04 [0.84–1.24]	.25
Uric acid, mg/dL	5.9 [5.1–6.9]	6.5 [5.1–7.6]	6.0 [5.2–7.2]	.49
Total cholesterol, mg/dL	170 [139–185]	171.5 [145–203]	169 [134.5–199]	.40
LDL cholesterol, mg/dL	97 [85–125]	104 [81–135]	103 [75–133]	.39
HDL cholesterol, mg/dL	42 [38–49]	45 [38–53]	41 [36–49]	.055
Triglyceride, mg/dL	86.0 [72–108]	93 [72–126]	97 [76–138]	.69

Major bleeding was observed in 88 (12.5%) and minor bleeding in 74 (10.5%) patients in the study group. The median time to bleeding was 135 days. Target vessel revascularization was performed in 48 patients (6.8%), and stroke was observed in 64 patients (9.1%). The mortality rate of the patients in the study group was 14.8%. The clinical features and other prognostic implications of bleeding after ACS are detailed in Table 3.

**Table 3:** Poor clinical outcomes and times of the groups at 1-yr follow-up.

		Charlson Comorbidity Index (CCI)			
	Overall (n = 704)	CCI: 0 (n = 156)	CCI: 1 (n = 266)	CCI ≥ 2 (n = 282)	*P*
Major bleeding, n (%)	88 (12.5)	10 (6.4)	36 (13.5)	42 (14.9)	.17
Minor bleeding, n (%)	74 (10.5)	20 (12.8)	24 (9.0)	30 (10.6)	.68
Major bleeding time, d	145.4 ± 90.1	159.5 ± 83.2	168.2 ± 104.9	122 ± 73.9	.27
Target vessel revascularization	48 (6.8)	2 (1.3)	20 (7.5)	26 (9.2)	.07
Target vessel revascularization time, d	160.6 ± 115.3	94	150 ± 119.8	173.9 ± 118.6	.76
Acute ischemic stroke.	64 (9.1)	4 (2.6)	18 (6.8)	42 (14.9)	.005
Acute ischemic stroke time, d	108 ± 77.9	95.5 ± 64.3	93.5 ± 85.3	115.4 ± 78.2	.77
All-cause mortality	104 (14.8)	4 (2.6)	20 (7.5)	80 (28.4)	<.001
Mortality time, d	58.6 ± 64.2	97.5 ± 37.4	37.9 ± 49.4	61.9 ± 67.7	.39

As shown in Table 3, the groups were similar in terms of major and minor bleeding, time of bleeding, target vessel revascularization and time, time of stroke, and time of death (*P* > .05). Acute ischemic stroke development was significantly higher in patients in the CCI ≥ 2 group compared to the other 2 groups (*P* = .005). Mortality rates were found to be 28.4%, 7.5%, and 2.6% in patients with CCI ≥ 2, CCI 1, and CCI 0, respectively. The mortality rate of the CCI ≥ 2 group was significantly higher than those of the other 2 groups (*P* < .001).

**Figure 1.**
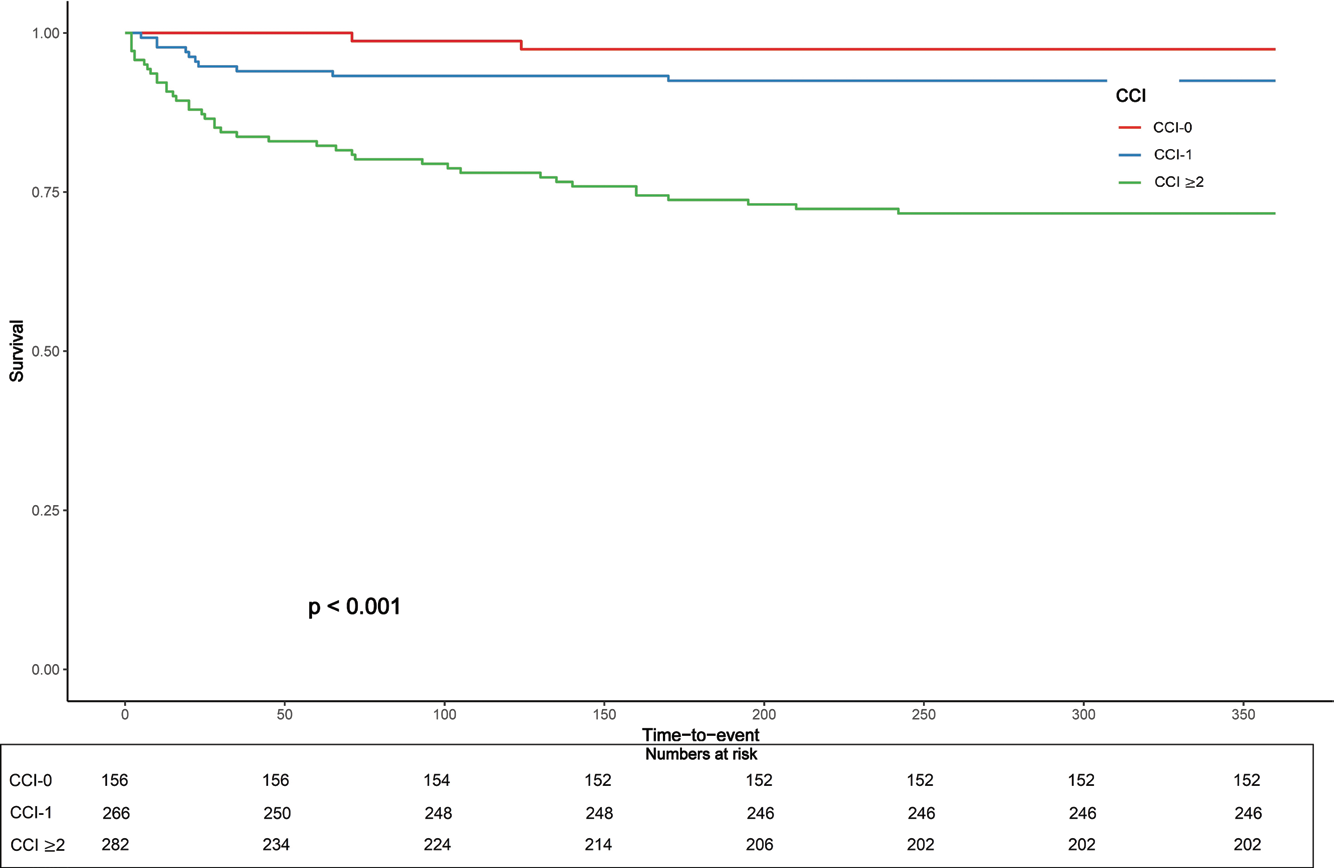
The Kaplan–Meier survival analysis revealed differences in the all-cause mortality rates between the CCI groups. CCI = Charlson comorbidity index.

The results of Cox regression analysis are presented in Table 4. The multivariate Cox proportional hazard regression model showed that CCI was an independent predictor for 1-year all-cause mortality (hazard ratio: 1.632; 95% confidence interval: 1.403–1.898; *P* < .001). The Kaplan–Meier survival analysis revealed differences in the all-cause mortality rates between the CCI groups at the 1-year follow-up shown in Figure 1.

**Table 4:** Cox regression analysis for the risk factors in predicting the all-cause mortality.

	Univariate	Multivariate
	HR (95% confidence interval)	HR (95% confidence interval)
BMI	0.970 (0.937–1.004, *P* = .08)	0.991 (0.955–1.029, *P* = .64)
LvEF	0.958 (0.934–0.982, *P* = .001)	0.995 (0.967–1.024, *P* = .73)
Grace score	1.018 (0.998–1.038, *P* = .08)	1.020 (0.999–1.042, *P* = .06)
CCI	1.659 (1.455–1.892, *P* < .001)	1.632 (1.403–1.898, *P* < .001)

CCI = Charlson comorbidity index, HR = hazard ratio, LvEF = Left ventricular ejection fraction.

## 4. Discussion

Our studied revealed the relationship between CCI, which is an indicator of comorbidity burden, and cerebrovascular accidents and all-cause mortality in a 1-year follow-up of elderly patients who underwent percutaneous coronary intervention due to ACS.

Elderly patients are more fragile due to the increasing burden of comorbidity with age, and scoring systems, which are indicators of the comorbidity burden based on this frailty, can be used to predict patients’ prognosis. CCI, developed in 1987, is still used as an indicator of the comorbidity burden.^[8]^ While studies have shown the prognostic value of the CCI after ACS, our study stands out due to the mean age of the patients and the number of participants.^[3,9]^

The CCI includes non-cardiovascular comorbidities, such as chronic liver diseases, malignant neoplasm, and lymphoma. Previous studies have shown that the prognosis of patients with cardio vascular diseases worsens as the comorbidity burden increases.^[10]^ Similarly, in our study, patients with 2 or more comorbidities had worse survival rates at the 1-year follow-up. As the number of comorbidities increases, patients become more vulnerable and fragile. It has been shown that CCI, which is an indicator of comorbidity burden, has prognostic importance not only for cardiovascular diseases but also for cerebrovascular diseases, trauma, and infection.^[11–13]^

Previous studies have demonstrated that the CCI is important both in the short term (e.g., in-hospital mortality) and in the long term (e.g., 2-year mortality).^[14,15]^ The GRACE (Global Registry of Acute Coronary Events) score is used to predict in-hospital and 6-month mortality in patients with ACS.^[15]^ However, comorbidities are ignored in the GRACE score. In our study, although there was no significant difference between the CCI groups in terms of GRACE scores, a significant difference was observed in mortality rates. This shows that apart from the GRACE score, the burden of comorbidity and comorbidity scores are also important in terms of mortality. A previous study showed that the CCI was not superior to the GRACE score at the 2-year follow-up and made no additional contribution.^[15]^ However, the fact that the participants in that study were younger than those in our study and that patients who did not receive invasive treatment were excluded in this study due to their poor overall prognosis, probably due to the high comorbidity burden, may have caused this difference. Comorbidity indices are more important for these patients, as the comorbidity burden increases with age, which makes elderly patients more vulnerable and fragile. Another study showed that the CCI added to the GRACE risk prediction index can be useful in predicting patient prognoses.^[16]^

In our study, as the comorbidity burden of patients who underwent percutaneous coronary intervention due to ACS increased, more cases of acute ischemic stroke were observed during the follow-up period. In previous studies, as in our study, it has been shown that more acute ischemic stroke is observed in patients as the comorbidity burden increases.^[14]^ In our study, target vessel revascularization was higher in the patient group with a high comorbidity burden at the 1-year follow-up, although the difference was not statistically significant. The reason for the lack of a significant difference may be that the patients in our study felt less angina because they were older, or they did not feel exertional angina due to their reduced daily activities.

Further, no significant differences were found in major and minor bleeding. The fact that patients with more comorbidities are less mobile causes them to be exposed to less trauma. Moreover, they may have applied to the hospital less because of minor bleeding due to their immobility.

Our study has some limitations. First, some comorbidities (e.g., AIDS) occur in low frequencies in the general population as well as in the study population, which consisted of elderly patients. Another limitation is the underreporting of secondary diagnoses, as the data were obtained from the hospital registry system since this was a retrospective study.

## 5. Conclusion

For elderly patients, frailty is a major concern, and the biggest cause of frailty in elderly patients is the comorbidity burden, which increases with age. Previous studies have shown that the patient prognosis will be worse as the comorbidity burden increases. Although there was no difference GRACE scores between the different CCI groups in our study, patients with high CCI scores had significantly higher rates of mortality and acute ischemic stroke at the 1-year follow-up. CCI was an independent predictor for 1-year all-cause mortality. CCI may contribute to treatment and follow-up management, as it indicates a poor prognosis in elderly patients who have undergone percutaneous coronary intervention.

## Acknowledgments

All individuals who contributed to this publication have been included as authors. The authors did not receive support from any organization for the submitted work.

## Author contributions

Conceptualization: Ahmet Balun.

Data curation: Ahmet Balun, Alkame Akgümüş.

Formal analysis: Ahmet Balun, Alkame Akgümüş.

Investigation: Ahmet Balun, Alkame Akgümüş.

Methodology: Ahmet Balun.

Resources: Ahmet Balun, Alkame Akgümüş

Software: Ahmet Balun.

Supervision: Ahmet Balun, Alkame Akgümüş.

Validation: Ahmet Balun.

Visualization: Alkame Akgümüş.

Writing – original draft: Ahmet Balun, Alkame Akgümüş

Writing – review & editing: Ahmet Balun, Alkame Akgümüş

## References

[1] Jiménez-Méndez C Díez-Villanueva P Alfonso F. Non-ST segment elevation myocardial infarction in the elderly. Rev Cardiovasc Med. 2021;22:779–86. 34565076 10.31083/j.rcm2203084

[2] Canivell S Muller O Gencer B. Prognosis of cardiovascular and non-cardiovascular multimorbidity after acute coronary syndrome. PLoS One. 2018;13:e0195174. 29649323 10.1371/journal.pone.0195174 PMC5896917

[3] Radovanovic D Seifert B Urban P. Validity of Charlson comorbidity index in patients hospitalised with acute coronary syndrome. Insights from the nationwide AMIS Plus registry 2002-2012. Heart. 2014;100:288–94. 24186563 10.1136/heartjnl-2013-304588

[4] Formiga F Moreno-Gonzalez R Chivite D. High comorbidity, measured by the Charlson comorbidity index, associates with higher 1-year mortality risks in elderly patients experiencing a first acute heart failure hospitalization. Aging Clin Exp Res. 2018;30:927–33. 29124524 10.1007/s40520-017-0853-1

[5] Jiménez Caballero PE López Espuela F Portilla Cuenca JC. Charlson comorbidity index in ischemic stroke and intracerebral hemorrhage as predictor of mortality and functional outcome after 6 months. J Stroke Cerebrovasc Dis. 2013;22:e214–8. 23352682 10.1016/j.jstrokecerebrovasdis.2012.11.014

[6] Zhou S Zhang XH Zhang Y. The age-adjusted Charlson comorbidity index predicts prognosis in elderly cancer patients. Cancer Manag Res. 2022;14:1683–91. 35573259 10.2147/CMAR.S361495 PMC9091471

[7] Kikkert WJ van Geloven N van der Laan MH. The prognostic value of bleeding academic research consortium (BARC)-defined bleeding complications in ST-segment elevation myocardial infarction: a comparison with the TIMI (Thrombolysis In Myocardial Infarction), GUSTO (Global Utilization of Streptokinase and Tissue Plasminogen Activator for Occluded Coronary Arteries), and ISTH (International Society on Thrombosis and Haemostasis) bleeding classifications. J Am Coll Cardiol. 2014;63:1866–75. 24657697 10.1016/j.jacc.2014.01.069

[8] Charlson ME Carrozzino D Guidi J. Charlson comorbidity index: a critical review of clinimetric properties. Psychother Psychosom. 2022;91:8–35. 34991091 10.1159/000521288

[9] Sanchis J Soler M Núñez J. Comorbidity assessment for mortality risk stratification in elderly patients with acute coronary syndrome. Eur J Intern Med. 2019;62:48–53. 30711360 10.1016/j.ejim.2019.01.018

[10] Beska B Mills GB Ratcovich H. Impact of multimorbidity on long-term outcomes in older adults with non-ST elevation acute coronary syndrome in the North East of England: a multi-centre cohort study of patients undergoing invasive care. BMJ Open. 2022;12:e061830. 10.1136/bmjopen-2022-061830 PMC9330324 35882457

[11] Jiang L Chou ACC Nadkarni N. Charlson comorbidity index predicts 5-year survivorship of surgically treated hip fracture patients. Geriatr Orthop Surg Rehabil. 2018;9:215145931880644. 10.1177/2151459318806442 PMC6249653 30479849

[12] Liu H Wu X Cao J. Effect of comorbidity assessed by the Charlson comorbidity index on the length of stay and mortality among immobile hemorrhagic stroke patients younger than 50 years. Front Neurol. 2020;11:1–6. 32625159 10.3389/fneur.2020.00487 PMC7314940

[13] Bariş SA Boyaci H Akhan S. Charlson comorbidity index in predicting poor clinical outcomes and mortality in patients with COVID-19. Turk Thorac J. 2022;23:145–53. 35404247 10.5152/TurkThoracJ.2022.21076 PMC9449884

[14] Zhang F Bharadwaj A Mohamed MO. Impact of Charlson co-morbidity index score on management and outcomes after acute coronary syndrome. Am J Cardiol. 2020;130:15–23. 32693918 10.1016/j.amjcard.2020.06.022

[15] Hautamäki M Lyytikäinen LP Mahdiani S. The association between charlson comorbidity index and mortality in acute coronary syndrome—the MADDEC study. Scand Cardiovasc J. 2020;54:146–52. 31775530 10.1080/14017431.2019.1693615

[16] Erickson SR Cole E Kline-Rogers E. The addition of the Charlson comorbidity index to the GRACE risk prediction index improves prediction of outcomes in acute coronary syndrome. Popul Health Manag. 2014;17:54–9. 23965044 10.1089/pop.2012.0117

